# The well-being of young leaders: demands and resources from a lifespan perspective

**DOI:** 10.3389/fpsyg.2023.1187936

**Published:** 2023-05-11

**Authors:** Hanna Irehill, Robert Lundmark, Susanne Tafvelin

**Affiliations:** Department of Psychology, Umeå University, Umeå, Sweden

**Keywords:** young leaders, lifespan, well-being, work environment, JD-R

## Abstract

Building on the job demand resources (JD-R) model, we examined the experience of work environment and well-being among young leaders in a two-wave survey study of 1,033 leaders within the private sector in Sweden. Our results reveal that young leaders report higher levels of burnout and lower rates of vigor compared to older colleagues. Further, they appraise demand and resources differently, perceiving higher emotional demands and less organizational support, and they seem to struggle with the leader role, seeing it as unclear and conflicting. Our findings underline the necessity of viewing the leader role from a lifespan perspective as well as considering age-specific aspects in the JD-R model. In practice, we urge organizations to improve prerequisites for young leaders by providing support and role clarifications to prevent impaired well-being and improve retention. By bringing leadership and lifespan studies together, we aim for a better understanding of what specific prerequisites young leaders need to thrive in the leader role thus showing how age matters and bring the field of research forward.

## 1. Introduction

Well-being is a fundamental aspect of working life and a key aspect to consider when aiming to understand what makes us thrive at the workplace (Danna and Griffin, 1999). Abundant empirical evidence has stated the far-reaching implications of leader well-being on follower well-being, leader behavior and organizational outcomes (Kaluza et al., 2020). However, our understanding as to whether leader well-being differ with respect to age is almost absent (Zacher et al., 2015), specifically regarding young leaders. A recent empirical study revealed that young leaders experience the work environment as stressful with lack of support (Larsson and Björklund, 2020). In addition, the interest in leader roles among young individuals seems to vary; some view leader roles as far too demanding and difficult to balance with private life (Larsson and Björklund, 2020). As a result, the well-being of young leaders may be affected (Salminen et al., 2014). If young leaders experience poor well-being and rule out leader roles as a career choice, who will be the leaders of tomorrow?

Despite this worrying trend, there is a lack of knowledge about how young leaders experience their work environment and well-being. Although still in its infancy, a lifespan perspective on leader roles has been proposed (Zacher et al., 2015) which in effect mean a conceptualization of age including age-related gains and losses rather than just the chronological number itself. Thus, approaching age from a lifespan perspective could provide a more comprehensive understanding about the situation for young leaders since it considers age-related processes and development (Baltes, 1987; de Lange et al., 2010).

The aim of the present study is to investigate how young leaders experience their work environment and well-being from a lifespan perspective. Leaning on the job demand resources theory (JD-R; Demerouti et al., 2001), the present study contributes to the field in three important ways. First, we contribute to the emerging field of lifespan and leadership research by adding new knowledge about how young leaders experience their work environment and well-being. A lifespan perspective considers developmental factors such as benefits and challenges in various stages of life, enabling a more sophisticated understanding of what it is like being young in a leader role (Rudolph et al., 2018). Second, by applying a lifespan perspective on the JD-R model we shed light on possible age aspects considering experiences of demand and resources and its associations to well-being. Because the JD-R model can explain how predictors in the work environment may affect well-being outcomes, these pathways are relevant to consider (Bakker et al., 2014). Suggestions have been made that demands and resources might predict health differently based on age (Haley et al., 2014), but to our knowledge, the JD-R model has not been viewed from a lifespan perspective before. This is important because differences in experiences might call for adjustments in the work environment that ultimately could improve leader well-being.

## 2. Literature review and hypothesis development

### 2.1. Leaders’ well-being and work environment

Well-being is a broad concept that takes the whole person into consideration, including subjective experiences of physical and psychological health and functioning (Danna and Griffin, 1999). Psychological well-being includes affective and cognitive processes that may be related to both work and non-work domains and can lead to positive or negative outcomes (Warr, 2013). Leaders’ well-being has not been studied nearly as much as that of followers (Barling and Cloutier, 2017), nor what conditions they face in the workplace that might affect how they feel (Nielsen and Taris, 2019). Two contrasting views on leader well-being have been suggested, in which the leader role might affect well-being in both positive and negative directions through indirect effects of demand and control (Li et al., 2018). For example, Nyberg et al. (2015) found that leaders experienced higher demands than non-leaders but also reported access to more resources, such as influence. However, recent reports reveal that leader well-being, independent of age, has deteriorated in recent years (Previa, 2019), which raises questions about how the work environment is experienced.

The JD-R model (Demerouti et al., 2001) states that resources may serve as a buffer to high demands and hence prevent poor health and enhance well-being. Due to its flexibility, taking context-specific resources and demands into consideration, the JD-R model has been commonly used (Schaufeli and Taris, 2014). In short, the JD-R model presents two pathways; the health impairment process states that high demands yield strain and health debilitation, while the motivational process describes how access to resources enhances motivation and performance (Bakker and Demerouti, 2007). Thus, the JD-R model can explain both negative and positive aspects of occupational well-being, such as processes that lead to work engagement and feelings of vigor or those leading to exhaustion or burnout (Bakker et al., 2014). Although leadership has been integrated in the JD-R model (Schaufeli, 2015), a lifespan perspective is still missing. Consequently, the present study is intended to view experiences of demands, resources and well-being of young leaders.

### 2.2. Leaders’ work environment from a lifespan perspective

When applying a lifespan perspective to leader roles, either the entire adult lifespan or specific age-related stages should be considered (Rudolph et al., 2018). Liu et al. (2021) suggests how challenges and benefits at various stages through the lifespan affect the leadership development process. Young leaders in “The self and opportunity related stage” captures 18–30 year olds in a period of life that usually precedes family building and care for elderly relatives (Liu et al., 2021). This period of the lifespan has also been theorized and modelled for young workers in general (Super, 1980). At this stage, young leaders may benefit from their self-motivation to seek new experiences and challenges that may result in growth (Liu et al., 2021), an approach that could be explained by the socioemotional selectivity theory (SST; Carstensen et al., 1999). According to SST, young individuals perceive time as open-ended and are prone to seek progression and development. Older individuals have a more limited time perspective and are more motivated by emotion-related goals from a shorter perspective (Lang and Carstensen, 2002). As possibilities for development arise during “the self and opportunity related stage” (Liu et al., 2021), this period of life also brings instability and change due to vocational decisions, and social relationships that might affect well-being (Akkermans et al., 2009; Liu et al., 2021). In fact, it has been suggested that young individuals are exposed to high demands and insufficient resources at work and in their private lives simultaneously (Demerouti et al., 2012). To meet demands, the use of action regulation strategies such as selection, optimization and compensation (SOC; Baltes et al., 1999) has proved helpful in terms of maintaining well-being despite high demands (Freund and Baltes, 2002). *Selection* refers to decisions about what goals to pursue, as young individuals tend to select goals that lead toward enhanced development, whereas loss-based selection refers to maintaining function and is more prevalent at higher ages. *Optimization* consists of the strategies used to apply resources to meet goals, and *compensation* refers to the process of mitigating a lack of resources (Cadiz et al., 2019). However, the use of SOC strategies is likely increased and refined over time due to accumulation of experience of various situations in life (Freund and Baltes, 2002), leaving young individuals with less readiness to deal with potential challenges of work life. Indeed, lack of experience and coping skills to face everyday situations at work might cause higher levels of stress (Duchscher, 2009). Besides being young, entering a leader role has been associated with stress and tension, a taxing experience that effect well-being negatively (Fletcher and French, 2021). However, the authors found that leader well-being improved over time because of possibilities to develop a richer and more positive self-concept within the leader role (Fletcher and French, 2021). Thus, experiences of the leader role seem dual in its nature over time and experiences of being young in the “self and opportunity related stage” entails advantages and disadvantages due to developmental prerequisites and contextual factors in life. In other words, the combination of being both young and inexperienced regarding the leader role pose potential challenges. To fully understand the well-being of young leaders, we need to gain insight into how developmental aspects change with age, such as strategies, motives, and psychological states (Scheibe and Zacher, 2013), as well as specific life roles and phases (Kanfer and Ackerman, 2004).

### 2.3. Job demands-triggers of the health impairment process

The JD-R model defines demands as physical, psychological, social, or organizational aspects that require sustained effort and skill to meet and are therefore associated with a certain cost (Bakker and Demerouti, 2007). These demands can for instance be quantitative, emotional, or include role-conflict (Schaufeli and Taris, 2014). Quantitative demands refer to the amount of work, the distribution of workload, and time pressure (Pejtersen et al., 2010). Several studies have concluded that the leader role implies high quantitative demands in general (Nyberg et al., 2015), and it has recently been suggested that young workers may experience strain due to higher cognitive demands with reference to time pressure (Abbasi et al., 2019). Although young leaders have a lot of energy in their quest for growth and increased knowledge (de Lange et al., 2010) several requirements are placed on young leaders. Acclimatizing to a new setting, understanding and fulfilling expectations would require a high effort, particularly with respect to the limited time given to be functional in the leader role (Fletcher and French, 2021). Consequently, young leaders would most likely experience high quantitative demands.

Regarding leadership, emotions play an important role in several ways (Rosing and Jungman, 2019). For instance, recognizing and addressing emotions is important in the process of building relationships with followers and gaining acceptance as a leader (Humphrey, 2002), where emotion-related abilities could be seen as an advantage. Emotional demands refer to the degree of negative emotional strain at work (Pejtersen et al., 2010), including the consideration of one’s own emotional demands, employees’ affective states, and context-specific situations that might be emotionally demanding. According to SST (Carstensen et al., 1999), older workers seem to value emotionally positive states and meaningful social relationships as well as to avoid negative affective experiences to a higher extent than younger counterparts. This might help them maintain emotional well-being and fulfil emotional demands at work (Kanfer and Ackerman, 2004; Scheibe and Zacher, 2013). Because emotion regulation abilities develop with age (Kim and Kang, 2017), young leaders potentially have less resources to manage emotional demands.

The leader role includes a great variety of duties and responsibilities ranging from performing tasks at hand to establish relationships with followers and peers, indicating an urge to navigate and prioritize (Mumford et al., 2007). Role conflict refers to the experience of contradictory expectations, conflicts between preferences and given instructions, and inconsistent requests (Pejtersen et al., 2010). A meta-analysis by Ng and Feldman (2010) revealed that experiences of role conflict seemed to decrease with age, indicating age differences in leaders’ experiences of role conflict. A previous study confirmed that the use of SOC resulted in maintained function despite high demands (Moghimi et al., 2019). However, it has been suggested that access to selection, optimization, and compensation of resources might need to be reframed in a new context (Zacher and Frese, 2011), and with less experience, it is probably more difficult to judge which tasks to prioritize. Thus, drawing on SST and SOC we suggest that young leaders experience higher demands that older counterparts.

*Hypothesis* 1: Young leaders experience (a) higher quantitative demands, (b) higher emotional demands and (c) higher role conflict compared to their older colleagues.

### 2.4. Job resources—a part of the motivational process

Resources are defined as physical, psychological, social, or organizational aspects that reduce the effect of job demands and costs, aid in achieving work goals, and stimulate personal growth (Bakker and Demerouti, 2007). Role clarity, influence, social support, and organizational support are examples of resources (Schaufeli and Taris, 2014).

Role clarity refers to the experience of clear objectives, expectations, and responsibilities (Pejtersen et al., 2010). Some have argued that young leaders need clearer boundaries and support to know what is expected in the leader role (Buengeler et al., 2016), implying that the role is not clear enough. In situations where goals, demands, and resources are misaligned, SOC strategies are helpful but the usage of SOC does not peek in early adulthood (Freund and Baltes, 2002) leaving the young leader with less resources to handle this demand. Moreover, young leaders need to identify themselves in leader roles (Benjamin and O’Reilly, 2011) and live up to assigned obligations, suggesting parallel processes of exploration and role fulfilment, which presumably are neither clear nor straightforward.

Influence relates to possibilities of making impacts in the workplace, affecting the amount of work and forms of cooperation (Pejtersen et al., 2010). Young leaders might encounter difficulties obtaining acknowledgement from followers (Buengeler et al., 2016), as gaining affirmation as a leader can be comprehended as a social process (DeRue and Ashford, 2010). The young leader has to overcome assumptions that coincide with older age, such as higher competency, higher status, and more knowledge (Kearney, 2008). Previous research has concluded that age differences between followers and leaders may have negative consequences (Shore et al., 2003), which in turn may aggravate a young leader’s potential to influence. Hence, it is reasonable to assume that a young leader implicitly has less authority and, as a result, fewer possibilities to influence.

Social support from colleagues refers to the possibility to gain help and support from colleagues, who listen and devote time to social interaction (Pejtersen et al., 2010). Social support is perceived as an important resource independent of age (Truxillo et al., 2012), although the leader role is often associated with loneliness (Li et al., 2018). Previous studies suggest that social support from colleagues is particularly important for younger workers (Akkermans et al., 2009). However, young individuals mainly tend to interact with colleagues outside the organization and not engage as much in social interactions at work unless it involves feedback that can be used to build knowledge and networks for a longer perspective (Cadiz et al., 2019). Meanwhile, as older individuals highly value emotionally fulfilling relationships (Carstensen et al., 1999), their social behavior in the workplace would probably generate increased social support. Thus, engaging less in social interaction would most likely render less social support despite the need for it.

Perceived organizational support refers to the experience of an organization sincerely caring about individual opinions, well-being, and goals, as well as making support available (Eisenberger et al., 1997). A recent study revealed that gaining supervisor support reduced sickness absence in young (but not old) workers (Bouville et al., 2018), indicating that age might moderate the importance of this resource. Calls have been made that leaders in their early careers need role models to develop appropriate leader behaviors and interpersonal relationships (Benjamin and O’Reilly, 2011). Further, drawing on SST (Baltes et al., 1999), young leaders would probably benefit from organizational support in terms of developmental programs and mentoring due to their high potential for improvement. Hence, we assume that the need to gain organizational support among young leaders is high even if the availability is not necessarily present. In contrast, pathways of seeking support or social informal networks might not yet be established, which means that young leaders are dependent on the organizational support offered. Together, we believe that young leaders experience less resources than older leaders, drawing on SOC and SST we predict:

*Hypothesis* 2: Young leaders experience (a) less role clarity, (b) less possibilities to influence, (c) less social support from colleagues, and (d) less organizational support compared to their older colleagues.

### 2.5. Well-being in terms of vigor and burnout

In line with the health impairment process and the motivational process of the JD-R model (Bakker et al., 2014), two diverse outcomes of well-being will be investigated: vigor and burnout. Burnout includes being physically and emotionally exhausted (Pejtersen et al., 2010), whereas vigor is a state of high energy level, mental resilience at work, willingness to invest effort, and persistence in the face of difficulties (Bakker et al., 2014). Because of presumed higher demands, fewer resources, the well-being of young leaders should be impaired in terms of higher levels of burnout and less vigor compared to older colleagues, following the two processes of the JD-R model. For instance, an excessively high degree of emotional demands may lead to emotional exhaustion, which is a central aspect of burnout. Younger workers tend to report emotional exhaustion to a higher extent than older colleagues (Ng and Feldman, 2010), which might be explained by how stressful situations are appraised and available coping strategies (Scheibe and Zacher, 2013). As job demands have been recognized as the main cause of burnout (Bakker et al., 2014), similar reasoning could be applied to the remaining demands and their association with experiences of burnout for young leaders. In addition, lower access to resources equals inferior buffers against demands, which ultimately will affect well-being negatively for young leaders. According to the JD-R model and the motivational process, resources also contribute to engagement, motivation, and states of vigor. Thus, young leaders have access to fewer resources and consequently less vigor. We predict:

*Hypothesis* 3: Young leaders experience (a) more burnout and (b) less vigor than their older colleagues.

### 2.6. The relative influence of job demands and resources on well-being from a lifespan perspective

Due to their lack of experience and less developed coping strategies (Scheibe and Zacher, 2013), young leaders are expected to appraise demands and resources differently than their older colleagues. For instance, with age, mood fluctuations seem to decrease and abilities to regulate positive and negative emotions increase (Morgan and Scheib, 2014). In addition, it has been argued that preexisting levels of well-being may impact how demands and resources are appraised (de Lange et al., 2005). Young leaders face many challenges in several domains and thus might be more vulnerable to high demands, have less access to resources, resulting in stronger consequences on their well-being. Hence, we predict that the appraisal of demands and resources has a stronger impact on the well-being of young leaders compared to older leaders. Thus, we posit:

*Hypothesis* 4: The association between work factors; demands on well-being and resources on well-being, will be stronger for young leaders, resulting in (a) higher levels of burnout and (b) lower levels of vigor.

## 3. Methods

### 3.1. Sample and procedure

Leaders within the private sector in Sweden (*N* = 1,033) participated in this two-wave survey study. The sample was stratified and randomly selected from the Swedish occupational register and included leaders at companies with at least 10 employees. At the first time point, 2,000 leaders aged 19–29 and 2,000 leaders aged 30–65 were invited to participate. With an aim to catch early experiences of the leader role we adopted the definition of young leaders (18–30 years old) by Liu et al. (2021). The response rate, corrected after deleting respondents who no longer worked as leaders, was 14% (*n* = 259) among young leaders and 36% (*n =* 638) for older leaders. After 6 months, a follow-up survey was sent to those who answered the first time. The response rate among young leaders at follow-up was 32% (*n =* 82) and 63% (*n* = 385) for the older leaders. During the second wave of data collection an additional sample of 1,091 young leaders were invited to participate, due to the relatively low response rate. The additional sample were added to the baseline measure of young leaders since they only responded to the survey once. The corrected response rate for the sample of new young leaders was 14% (136). Consequently, there were 395 young leaders in the baseline group. At the first time point, the survey was sent by post; at follow up, email was added with the intention to reach out to a larger number of young leaders. Administration and compilation of the survey were completed in cooperation with Statistics Sweden (i.e., a government agency responsible for national statistics). The mean age among young leaders was 27.7 years (SD = 1.5) and older leaders had a mean age of 49.6 years (SD = 8.2). Regarding education, 44% of the young leaders reported university as the highest completed educational level, corresponding to 57% within the group of older leaders. In total, 407 (39%) of participants were women; within the group of young leaders, 201 (51%) were women. Among young leaders, 61% were first line managers and 39% had positions at middle- or senior-levels of management. Corresponding figures for older leaders were 44% at the first-line level and 56% on middle- or senior-positions. The younger leaders had been employed in their current positions for an average of 2.8 years (SD = 2.1) and had been working as leaders for 3.6 years (SD = 2.1). On average, the older leaders had been employed in their current positions for 8.6 years (SD = 7.5) and had been working as leaders for 14.4 years (SD = 9.1). An attrition analysis performed by SCB revealed that being a woman, married, or older; having been born in Sweden; or having a higher educational level was associated with a higher probability of answering our survey.

### 3.2. Measures

The present study investigated how leaders experience demands and resources at work and well-being over time. All variables were measured at baseline. Vigor and burnout were also measured at follow up to investigate health outcomes. Internal consistency for all scales is presented in Table 1.

**Table 1:** Descriptive statistics.

Construct	Latent mean	SD	1.	2.	3.	4.	5.	6.	7.	8.	9.	10.	11.
1. Quantitative D. T1	3.53	0.60	*ω* = 0.85										
2. Emotional D. T1	3.16	0.56	0.43	*ω* = 0.80									
3. Role conflict T1	3.01	0.52	0.50	0.55	*ω* = 0.68								
4. Role clarity T1	3.69	0.67	−0.29	−0.19	−0.45	*ω* = 0.77							
5. Influence T1	3.93	0.48	−0.33	−0.16	−0.27	0.48	*ω* = 0.70						
6. Social support T1	3.55	0.69	−0.36	−0.24	−0.32	0.37	0.43	*ω* = 0.73					
7. Pos T1	5.53	0.95	−0.32	−0.31	−0.50	0.52	0.59	0.55	*ω* = 0.88				
8. Burnout T1	2.71	0.87	0.45	0.51	0.40	−0.38	−0.35	−0.27	−0.37	*ω* = 0.88			
9. Vigor T1	5.81	0.97	−0.21	−0.27	−0.32	0.47	0.42	0.27	0.49	−0.56	*ω* = 0.88		
10. Burnout T2	2.75	0.79	0.37	0.48	0.36	−0.33	−0.29	−0.13	−0.33	0.76	−0.47	*ω* = 0.87	
11. Vigor T2	5.79	0.94	−0.23	−0.26	−0.29	0.41	0.33	0.18	0.40	−0.46	0.71	−0.57	*ω* = 0.88

Latent means, standard deviation, reliabilities, and latent variable correlations at baseline and follow up.

*N* = 1,033. Reliabilities (omega) are presented on the diagonal. All correlations were significant at level *p* < 0.001.

SD = standard deviation. Quantitative D = Quantitative demands. Emotional D = Emotional demands. Pos = perceived organizational support.

Quantitative demands were measured using a four-item scale from the Copenhagen Psychosocial Questionnaire (COPSOQ II; Pejtersen et al., 2010). An example item from this questionnaire is “Is your workload unevenly distributed so it piles up?” Answers were obtained on a five-point Likert scale ranging from 1 (“*never/hardly ever*”) to 5 (“*always*”).

Emotional demands were assessed through a four-item scale from COPSOQ II (Pejtersen et al., 2010). An example item is “Do you get emotionally involved in your work?” Responses were obtained on a five-point Likert scale ranging from 1 (“*never/hardly ever*”) to 5 (“*always*”).

Role conflict was measured by a four-item scale from COPSOQ II (Pejtersen et al., 2010). An example item is “Are contradictory demands placed on you at work?” Responses were given on a five-point Likert scale from 1 (“*to a very small extent*”) to 5 (“*to a very large extent*”).

Role clarity was measured by a three-item scale from COPSOQ II (Pejtersen et al., 2010). An example item is “Does your work have clear objectives?” Responses were obtained on a five-point Likert scale ranging from 1 (“*to a very small extent*”) to 5 (“*to a very large extent*”).

Influence was measured by a four-item scale from COPSOQ II (Pejtersen et al., 2010). An example item is “Do you have the opportunity to influence important decisions concerning your work?” Responses were obtained on a five-point Likert scale ranging from 1 (“*never/hardly ever*”) to 5 (“*always*”).

Social support from colleagues was assessed through a three-item scale from COPSOQ II (Pejtersen et al., 2010). An example item is “How often do you get help and support from your colleagues?” Responses ranged from 1 (“*never/hardly ever*”) to 5 (“*always*”) on a five-point Likert scale.

Perceived organizational support was measured through an eight-item scale, a shorter version of the Survey of Perceived Organizational Support (Eisenberger et al., 1997). An example item is “Help is available from my organization when I have a problem.” Responses ranged from 1 (“*strongly disagree*”) to 7 (“*strongly agree*”) on a seven-point Likert scale.

Burnout was measured by a four-item scale from COPSOQ II (Pejtersen et al., 2010). An example item is “How often have you felt worn out?” Answers were obtained through a five-point Likert scale with options ranging from 1 (“*not at all*”) to 5 (“*all the time*”).

Vigor was measured by a three-item scale from the shortened version of the Utrecht Work Engagement Scale (Schaufeli et al., 2006). An example item is “When I get up in the morning, I feel like going to work.” Response categories ranged from 1 (“*never*”) to 7 (“*every day*”).

### 3.3. Statistical analyses

Analyses were performed with Mplus software, Version 8 (Muthén and Muthén, 2017) and all models were estimated with maximum likelihood. Initially, a confirmatory factor analysis was performed for every construct separately to investigate reliability and start building a measurement model. Secondly, a confirmatory factor analysis was conducted for the measurement model in full and for each group (younger and older leaders). In the next step, measurement invariance was examined as a prerequisite to make group comparisons (Vandenberg and Lance, 2000). In addition, measurement invariance was also examined between the new selection of young leaders and those from the first time point to ensure that the two groups could be merged. To evaluate measurement invariance, the models were compared by fit indices at each step. Between configural and scalar, invariance was supported across groups when the Δ root mean square of approximation (ΔRMSEA) < 0.015, Δ comparative fit index (ΔCFI) < 0.01, and the Δ standardized root mean square residual (ΔSRMR) < 0.03 moving from configural to metric and when ΔSRMR <0.01 moving from metric to scalar, in line with recommendations (Chen et al., 2008).

To evaluate our hypotheses, multigroup analyses and structural equation modelling were employed. In the multigroup analysis, latent mean differences between younger and older leaders were investigated at baseline by assessing the critical ratio (CR) index. A CR value equal to or greater than 1.96 was considered a significant difference in latent means (Teo, 2015). To assess the relationships between work factors and their association with well-being over time, several structural models were applied. Analyses were performed with one predictor at a time on each outcome separately. Thus, each construct at baseline (e.g., emotional demands) was regressed on the outcome at follow up (burnout or vigor). Analyses were made for young and older leaders separately. A Wald test was conducted to detect whether there were significant differences between the groups. Model fit was assessed through several model fit indices, including a Chi-square fit index (*χ*^2^), RMSEA, SRMR, and CFI. In line with recommendations, acceptable fit was assumed when SRMR and RMSEA < 0.08 and CFI > 0.90. To specify an excellent fit, cut-off criteria were more restrictive (SRMR and RMSEA < 0.08, CFI > 0.95; Bentler, 2007).

## 4. Results

Descriptive statistics, correlations, and reliability measures are presented in Table 1. Items loaded as expected on their respective factor, and the latent variables correlated significantly (*p* < 0.001) in the expected direction.

### 4.1. Test of hypotheses

Before testing our hypotheses, we examined our measurement model, which showed acceptable fit: *χ*^2^(847) = 2,576, *p* < 0.001, RMSEA = 0.04, SRMR = 0.05, CFI = 0.91. Next, the measurement invariance at a scalar level was supported between groups, between the new sample of young leaders and young leaders at baseline, at the scalar level (see Table 2). Thus, through further analysis, a comparison of latent means was enabled as was the merging the two groups of young leaders.

**Table 2:** Multigroup invariance.

Models	Chi square	df	RMSEA	SRMR	CFI	Model comparison	ΔRMSEA	ΔSRMR	ΔCFI
*Overall Model*									
Configural	3506	1680	0.05	0.06	0.91				
Metric	3571	1713	0.05	0.06	0.91	Configural vs. Metric	0.00	0.00	0.00
Scalar	3702	1746	0.05	0.06	0.90	Metric vs. Scalar	0.00	0.00	0.01
*Model 2*									
Configural	1996	1186	0.06	0.07	0.89				
Metric	2061	1214	0.06	0.07	0.88	Configural vs. Metric	0.00	0.00	0.01
Scalar	2096	1242	0.06	0.08	0.88	Metric vs. Scalar	0.00	0.01	0.00

*Overall Model*: Includes all variables at T1and at T2 Vigor and Burnout. N = 1,029. Young leaders (*n* = 392) Older leaders (*n* = 637).

Items correlate over time. *Model 2:* Additional sample of young leaders (*n* = 136) and first edition young leaders (259) at T1.

RMSEA = Root mean square of approximation. SRMR = standardized root mean square residual. CFI = Comparative fit index.

Our hypothesis that young leaders experience quantitative demands to a greater extent than older leaders (Hypothesis 1a) was not supported; see Table 3. However, young leaders experienced significantly higher levels of emotional demands (*M*_young_ = 3.28, *M*_older_ = 3.09, Δ = 0.19, CR = −4.11), in line with Hypothesis 1b. As for the experience of the leader role, young leaders experienced role conflict to a higher extent (*M*_young_ = 3.14, *M*_older_ = 2.93, Δ = 0.21, CR = −4.07), and they reported lower levels of role clarity (*M*_young_ = 3.62, *M*_older_ = 3.73, Δ = −0.11, CR = 2.65), in support of Hypotheses 1c and 2a. Regarding influence, no statistically significant difference was found between the groups; thus, Hypothesis 2b was not supported. Further, contrary to Hypothesis 2c, young leaders did not report significantly less social support than older leaders. On the other hand, young leaders perceived less organizational support (*M*_young_ = 5.34, *M*_older_ = 5.65, Δ = −0.31, CR = 2.01), in line with Hypothesis 2d. Regarding experiences of well-being, Hypotheses 3a and 3b were supported: Young leaders experienced higher levels of burnout (*M*_young_ = 2.99, *M*_older_ = 2.53, Δ = 0.46, CR = −8.31) and lower levels of vigor compared to their older colleagues (*M*_young_ = 5.61, *M*_older_ = 5.93, Δ = −0.32, CR = 5.12).

**Table 3:** Multigroup analysis.

Construct	Young leaders (mean)	Older leaders (mean)	Delta (Δ)	CR
Quantitative demands	3.47	3.57	−0.1	1.24
Emotional demands	3.28	3.09	0.19	**−4.11**
Role conflict	3.14	2.93	0.21	**−4.07**
Role clarity	3.62	3.73	−0.11	**2.65**
Influence	3.92	3.93	−0.01	−0.40
Social support	3.61	3.52	0.09	−1.71
pos	5.34	5.65	−0.31	**2.01**
Burnout	2.99	2.53	0.46	**−8.31**
Vigor	5.61	5.93	−0.32	**5.12**

Comparison of latent means at baseline.

CR = critical ratio. CR values in bold > 1.96 equals *p* < 0.05.

Young leaders (*n* = 392), older leaders (*n* = 637).

Pos = perceived organizational support.

We found no support for Hypothesis 4a, which suggested that associations between work factors and burnout would be stronger for young leaders. However, three significant differences between groups were found. The impact of role clarity (Wald = 3.70, *p* = 0.05), social support (Wald = 5.91, *p* = 0.05), and perceived organizational support (Wald = 4.29, *p* = 0.05) on burnout differentiated between groups where the lack of these resources had a stronger association to burnout at the second time point for older leaders compared to young leaders. No differences were found between the groups for how quantitative demands, emotional demands, and role conflict affected burnout over time. In contrast to Hypothesis 4b, no significant difference was found between groups regarding the associations between work factors and vigor. Wald tests and parameter estimates are presented in Table 4.

**Table 4:** Associations between work factors and well-being, differences between younger- and older-leaders.

	Burnout Time two			Vigor Time two		
Time one predictors	Young β	Old Β	Wald Value	Young β	Old β	Wald Value
Quantitative demands	0.33**	0.35***	0.01	−0.32**	−0.19***	1.68
Emotional demands	0.38***	0.45***	0.57	−0.29**	−0.20***	0.79
Role conflict	0.19	0.33***	0.53	−0.11	−0.26***	0.28
Role clarity	−0.12	−0.35***	**3.70**	0.41***	0.38***	0.22
Influence	−0.19	−0.34***	1.23	0.37**	0.29***	0.59
Social support	0.18	−0.18**	**5.91**	0.06	0.21***	0.66
Perceived org. Support	−0.11	−0.36***	**4.29**	0.40***	0.40***	0.03

^***^p < 0.001, ^**^p < 0.01, Wald values in bold *p* < 0.05. Wald test: Differences between young leaders (*n* = 392) and older leaders (*n* = 637). Measures performed at scalar level, with one predictor at a time on each outcome separately. Pos = perceived organizational support.

## 5. Discussion

The aim of this study was to investigate how young leaders in the “self and opportunity related stage” experience their work environment and well-being from a lifespan perspective. Although the scope of the study is focused upon young leaders (i.e., 18–30), using lifespan as a point of departure can facilitate our understanding of why young leaders may be in an extra vulnerable position compared to older counterparts. Our results revealed that young leaders report poorer well-being, higher emotional demands, less organizational support and seem to struggle with the leader role in aspects of perceived higher role conflict and less role clarity compared to older leaders. Regarding associations between work factors and well-being over time, only 3 of 14 analyses revealed differences between groups, indicating that all leaders appraised the overall work environment in a similar way.

A comparison of the levels of quantitative demands showed that Hypothesis 1a was not supported, suggesting that young and older leaders perceive their workloads as similar. However, young leaders experienced emotional demands to a higher extent, in support of Hypothesis 1b. These results are in line with SST, suggesting that young leaders focus less on positivity maintenance (Scheibe and Zacher, 2013) and have less-developed coping strategies (Morgan and Scheib, 2014). As a result, young leaders may face increased difficulty handling emotional demands and would probably appraise them as more stressful (Doerwald et al., 2016). However, the downside of a positive orientation is that it might lead to a failure to recognize problematic situations and might induce avoidant behaviors that could have negative consequences for the work environment (Reed and Carstensen, 2012). Further research is needed on the plausible consequences of emotional development during the lifespan. Our results highlight the leader role as subject to improvement, as young leaders experience both role conflict and lack of role clarity, supporting Hypotheses 1c and 2a. These findings could be explained by young leaders’ lack of access to fine-tuned SOC strategies to adapt and handle the leader role. However, suggestions have been made that the use of SOC strategies can be developed through deliberate practice in organizations as activation of personal resources could enhance capability to meet demands and engagement at work (Zacher et al., 2014). For young leaders this could for instance be to practice how to select and prioritize everyday tasks, optimize the way to work (time allocation) and how to compensate for age related inexperience (such as asking for help) Adding on this, a well-defined leader role could prove helpful to compensate for a lack of experience and less developed SOC strategies. The organizational socialization process may also be of specific importance for young leaders because it increases role understanding and reduces uncertainty (Bauer et al., 2007).

Regarding the comparison of resources, contrary to Hypothesis 2b, young leaders did not experience less influence compared to older leaders. A plausible explanation, based on SST, could be that young leaders at this stage are primarily self-focused and do not consider the wider perspective of influence to the same extent as older colleagues, nor do they know what to expect from that aspect of the work environment. Regarding social support, young leaders did not experience less social support from colleagues compared to older leaders, which contradicts Hypothesis 2c. However, they did perceive less organizational support, in line with Hypothesis 2d. It has been proposed that various workplace issues might call for different types of support, even if contextual factors of social support among leaders have received little attention (Lundqvist et al., 2018). In line with SST, young leaders are prone to seek feedback due to their motivation to learn and develop (Carstensen et al., 1999), which implies that young leaders may ask for help from colleagues when needed. Towards the organization, the young leader needs to fulfil expectations and prove capable, which might make it more difficult to judge when to seek support and in what matter. From a learning perspective, adequate support and feedback enable deliberate practice, indicating that people do not automatically learn from experience in itself (Day and Thornton, 2018), which further strengthens the importance of organizational support. Altogether, that could explain why young leaders do not experience a lack of social support from their colleagues (Hypothesis 2c) but from their organizations (Hypothesis 2d). Consequently, our results suggest that organizations need to make sure that support structures are available.

Young leaders reported poorer well-being in terms of more burnout and less vigor than older leaders, in support of Hypotheses 3a and 3b. This is not surprising, considering the previously reported imbalance between demands and resources, such as high emotional demands and low organizational support. Thus, our results are in line with the predictions of the two pathways of the JD-R model and highlight the importance of a lifespan perspective on leader well-being. Previous research explained how age was positively related to work engagement due to increased emotion regulation strategies at higher ages (Kim and Kang, 2017). Our results lead in the same direction, that is; developmental aspects such as the ability to regulate ones emotions may affect outcomes related to well-being implying that less developed strategies makes it more difficult to meet demands and thrive. By extension, novel information about the well-being of young leaders could contribute to what young individuals need to get a good start in their working lives, because young individuals are less acquainted to the challenges in working life (Akkermans et al., 2013).

The association between work factors and burnout was stronger for older leaders regarding role clarity, social support, and organizational support, contradicting Hypothesis 4a. This implies that if an older leader experiences, for instance, less role clarity, this will have a greater association to well-being in terms of burnout. Contrary to Hypothesis 4b, no differences were found between groups considering the association between work factors and vigor. Only three of 14 associations revealed significant differences between groups, suggesting that leaders respond to their work environments in similar ways, irrespective of age.

### 5.1. Implications for research

Our results make three important knowledge contributions. First, we adapt a lifespan perspective to leadership research and move the field forward by answering this call (Rudolph et al., 2018). As a part of a growing body of evidence (e.g., Doerwald et al., 2021), our results strengthen the need to apply a lifespan perspective to leader roles because leaders in different stages of life experience demands and resources differently, which has consequences on their well-being. In other words, age matters regarding the leader role. Beyond our results, recent studies have suggested that young leaders may benefit from using different behaviors than older colleagues to gain acceptance from their followers and be perceived as effective (Kearney, 2008; Buengeler et al., 2016). Thus, there are several arguments for why a lifespan perspective on leader roles is important in future research. Second, our results also contribute to the scarce field of research on leader well-being (Barling and Cloutier, 2017). Li et al. (2018) presented a dual pathway model predicting that the leader role is positively related to both high demands and job control, which indirectly relate to different well-being outcomes. A young leader faces the same pathways as their older colleagues, regarding their exposure to both demands and resources. However, our results reveal that young leaders experience demands and resources differently and that their well-being is impaired, and this indicates that age might moderate these relationships. Thus, a lifespan perspective on leader well-being may enhance our understanding and point towards the need for further research. To gain a more extensive insight of what it is like to be young in leader roles, future research could preferably be conducted through in-depth interviews to bring clarity to this void. Finally, to view the JD-R model from a lifespan perspective, we shed light on the age aspects regarding the work environment of leaders. Our results underline that the pathways of JD-R for the two groups correspond to each other, meaning that the work environment affects leaders equally, regardless of their stage of life, suggesting that none of the groups are more vulnerable than the other. Moreover, this finding further validates the JD-R model and how the two pathways affect well-being outcomes specifically. However, because leaders experience demands and resources differently, this part of the JD-R model could be considered age-specific.

### 5.2. Implications for practice

One way to counteract the effects of high demands is to provide resources. Young leaders experience the leader role as both conflicting and unclear, indicating that the role’s description might be subject to improvement. These efforts should strive to increase congruence between the actual role description and the daily work to enable young leaders to calibrate their expectations with organizational demands. By extension, perhaps the leader role could allow for changes over time, with a stepwise increase in responsibility corresponding to development and learning. In this process, lifespan-specific advantages could be utilized and in this respect the leader role adjusted leaving behind the “one size fits all” approach. Furthermore, organizations could provide support in a more deliberate and pronounced way (Day, 2010), for instance, by systematically following up with young leaders over time through a solid introduction that includes mentorship. A mentor would give the young leader access to implicit knowledge about the leader role (Bass, 1990). Presumably, new situations will appear, and different types of support will be needed throughout the process of growing into the leader role. With such an introduction, perhaps it would be possible for young leaders to overcome implicit gender thresholds and capture and maintain an interest in this career choice.

### 5.3. Limitations and future research

A number of limitations need to be taken into consideration. First, young leaders had a notably low response rate, which limit the generalizability of the results. However, it is not unusual to have low response rates from people of younger ages (Statistics Sweden, 2020). To improve the sample size of young leaders, all young leaders within the private sector in Sweden were invited to participate. In addition, reminders were sent out after every time point, because this has been positively associated with enhanced response rates (Blumenberg et al., 2019). Another explanation to the large attrition rate could be the hypothesized problem; young, dissatisfied leaders resign due to deficient prerequisites, a self-select out process that we cannot control for in the present study. Thus, this limitation was difficult to overcome and highlights the shortage of young leaders. Future research might consider complementary study designs or make other arrangements to increase people’s motivation to respond. Moreover, a larger sample of young leaders could allow analysis of levels of leadership. Without experience, young leaders would likely occupy leader roles at the first-line level. Surprisingly, in our sample, differences in levels of leadership were not remarkably different between younger and older leaders. Nevertheless, we recommend future research to investigate levels of leadership and tenure as a contextual factor. Second, our survey was based on self-reported ratings from the same source, enhancing the risk for common method variance with systematic errors related to measurement (Podsakoff et al., 2003). However, we were interested specifically in capturing the experiences of young leaders and limiting the risk for common method variance with the two-wave design. Furthermore, in line with recommendations (Podsakoff et al., 2003), the order of questions and constructs in the survey was mixed, and the validated scales provided a variety of answer options to reduce the risk of common method bias. Third, another possible limitation concerns the measurement interval. Few methodological guidelines are available on what may constitute an appropriate interval between time points (Timmons and Preacher, 2015). Because experiences of burnout seldom appear overnight and include both psychological and physical symptoms (Bakker et al., 2014), lagged effects might require a measure interval of at least 1 year (Ford et al., 2014). Thus, this reasoning could challenge the current interval and shed light on why we were not able to demonstrate any differences between groups. On the other hand, state-like experiences have also been suggested in which the individual may feel more or less burned out or engaged at work on certain days compared to others (Sonnentag et al., 2010), which further highlights the question of which measurement interval is most appropriate.

## 6. Conclusion

We conclude that young leaders experience higher demands and lower access to resources than older colleagues and hence report poorer well-being. In conclusion, age matters: Older and younger leaders might need different prerequisites to lead and feel well at work, pointing towards the importance of a lifespan perspective and further research bringing the fields of leadership and lifespan closer. From a wider perspective, our results imply the need to look at age in working life in a more sophisticated way and take advantage of natural gains and losses throughout the lifespan. Thus, the prerequisites of taking on the leader role could be more appropriately tailored with respect to age. To nurture the possibilities for young leaders to remain in leader roles, organizations need to develop supportive strategies that can balance demands and resources and thus reduce the risk of impaired well-being. For that matter, organizations need to prepare better and turn age differences into an advantage.

## Data availability statement

The raw data supporting the conclusions of this article will be made available by the authors, without undue reservation.

## Ethics statement

The studies involving human participants were reviewed and approved by Etikprövningsmyndigheten. The ethics committee waived the requirement of written informed consent for participation.

## Author contributions

HI wrote the draft of the manuscript, organized the data file and conducted the statistical analysis together with RL. ST provided important feedback on the draft. The data collection was conducted in cooperation between the authors and Statistics Sweden. HI, RL, and ST contributed to the conception, design of the study and were active with the finalization of the manuscript. All authors contributed to the article and approved the submitted version.

## Funding

This study was supported by Afa Insurance (Grant number 180262). Publication fees is covered by the library of Umeå University.

## Conflict of interest

The authors declare that the research was conducted in the absence of any commercial or financial relationships that could be construed as a potential conflict of interest.

## Publisher’s note

All claims expressed in this article are solely those of the authors and do not necessarily represent those of their affiliated organizations, or those of the publisher, the editors and the reviewers. Any product that may be evaluated in this article, or claim that may be made by its manufacturer, is not guaranteed or endorsed by the publisher.
